# 47 Application of a Novel AI-Augmented-Lung Ultrasound for Use in Smoke Inhalation Injury Triage

**DOI:** 10.1093/jbcr/iraf019.047

**Published:** 2025-04-01

**Authors:** John Kubasiak, Jeffrey Carter, Niknam Eshraghi, Cynthia Gregory, Caelan Thomas, Bryson Hicks, Jeffrey Shupp, Kenton Gregory

**Affiliations:** Loyola University Chicago; Louisiana State University Burn Center; Legacy Oregon Burn Center; Oregon Health & Science University; Oregon Health & Science University; Oregon Health & Science University; MedStar Washington Hospital Center; Oregon Health & Science University

## Abstract

**Introduction:**

Inhalation injury is present in 10-20% of all burn admissions. Clinical diagnosis, triage, and prognostication remains difficult and with poor accuracy given the heterogeneity of injury patterns. In the setting of a mass casualty incident, the ability to accurately diagnosis inhalation injury is critical to triage and resource allocation for patients with inhalation injury. As such we sought to test the feasibility of using handheld ultrasound to detect smoke inhalation lung injury (SILI) in patients with smoke inhalation.

**Methods:**

An observational survey study was conducted. Patients with a history of smoke inhalation were recruited from 6 ABA verified burn centers. The patients underwent serial lung ultrasound scanning, based on a 14-zone scan protocol, using a handheld, tablet-based, commercially available ultrasound device with a curvilinear (C5-2) transducer. Ultrasound videos were centrally stored, and blinded image analysis was performed by 2-3 reviewers from a panel of 11 physicians with expertise in lung ultrasound. Final clinical determination of clinical SILI negative, simple, or complex patient categorization was performed in a blinded fashion with chart review completed by three of the study PIs.

**Results:**

130 subjects were enrolled. 84 subjects whose first LUS scan was performed within 48 hours of smoke inhalation were included for this analysis. 65/84 were clinically identified as positive for SILI with the remaining 19/84 subjects negative for SILI. 91% of subjects with smoke inhalation had at least one abnormal lung feature. Of the 84 subjects, the mean score of pleural line abnormalities was greater in SILI+ vs. SILI- subjects (p = 0.02). When subjects with > 10% TBSA were excluded, the significance of pleural line abnormalities between SILI+ vs. SILI- subjects improved from p = 0.02 to p = 0.002. Within the SILI+ subjects, Mean Pleural abnormalities between clinically simple or complex SILI determinations were 0.77 and 0.56 respectively (P = 0.04). Between groups patients’ sex, age, body mass index, smoking history and admission carboxyhemoglobin were not statistically significant.

**Conclusions:**

The detection of SILI induced lung injury with bedside ultrasound is feasible. It can detect abnormal lung features, and the presence of lung features correlates with the clinical diagnosis of SILI.

**Applicability of Research to Practice:**

Results from this survey study demonstrates robust Lung Ultrasound Changes present in almost all patients with SILI. These findings also demonstrate the ease of use and potential for use for triage of smoke inhalation patients in mass casualty events, forest fires, or fires in municipalities. Future prospective studies are needed to define if early LUS detection of SILI can improve triage of patients.

**Funding for the Study:**

DoD - 75A50120C000097

